# Predominant Dietary Pattern Characteristics and Their Association with Obesity-Related Metabolic Phenotypes in Middle-Aged and Older Chinese Adults: Findings from a Nationwide Cross-Sectional Study

**DOI:** 10.3390/nu18081245

**Published:** 2026-04-15

**Authors:** Wenjing Yan, Weihua Dong, Xiaona Zhang, Qingqing Man, Rongzhen Li, Yun Luo, Panpan Huang, Xiangjie Yao, Lianlong Yu, Lili Chen, Jian Zhang, Pengkun Song, Gangqiang Ding

**Affiliations:** 1 NHC Key Laboratory of Public Health and Nutrition, National Institute for Nutrition and Health, Chinese Center for Disease Control and Prevention, Beijing 100050, China; 15890793674@163.com (W.Y.); dwh19861535785@163.com (W.D.); zhangxn@ninh.chinacdc.cn (X.Z.); manqq@ninh.chinacdc.cn (Q.M.); chenll@ninh.chinacdc.cn (L.C.); zhangjian@ninh.chinacdc.cn (J.Z.); 2 NHC Specialty Laboratory of Food Safety Risk Assessment and Standard Development, Guangdong Provincial Center for Disease Control and Prevention, Guangzhou 511430, China; yc07606@um.edu.mo (R.L.); luoybest@163.com (Y.L.); hpp545396861@163.com (P.H.); ncuspyyaoxiangjie@163.com (X.Y.); 3 Shandong Center for Disease Control and Prevention, Shandong Provincial Academy of Preventive Medicine, Jinan 250014, China; lianlong00a@163.com

**Keywords:** dietary patterns, metabolic health, obesity, middle-aged and older adults, cross-sectional study, China

## Abstract

**Background/Objectives:** To identify major dietary patterns among adults aged ≥45 years and examine their associations with metabolic health–obesity phenotypes. **Materials and Methods:** We analyzed 32,091 adults aged ≥45 years from the 2015 China Adults National Chronic Disease and Nutrition Surveillance. Diet was assessed using three consecutive 24 h dietary recalls, with household condiment weighing used to improve estimation of cooking oil and salt intake. Dietary patterns were derived using principal component analysis and categorized into quartiles. Multinomial logistic regression adjusted for energy intake and key sociodemographic/lifestyle factors to estimate odds of metabolically unhealthy non-obesity (MUNO), metabolically healthy obesity (MHO) and metabolically unhealthy obesity (MUO) versus metabolically healthy non-obesity (MHNO). **Results:** Four patterns with geographic variation were identified: (1) the Rice–Vegetable–Pork pattern; (2) the Fruit–Egg–Dairy pattern; (3) the Red Meat–Offal–Snack pattern; and (4) the Soybeans–Tubers–Grains pattern. Compared with Q1, Q4 of Pattern 1 was associated with lower odds of MHO (*OR* = 0.42, 95% *CI*: 0.38–0.46) and MUO (*OR* = 0.40, 95% *CI*: 0.36–0.44). Pattern 2 and Pattern 3 were associated with higher odds of MHO (Q4 vs. Q1: *OR* = 1.42 and 1.21) and MUO (*OR* = 1.36 and 1.14, all *p* < 0.001). Pattern 4 was inversely associated with MHO (*OR* = 0.85, 95% *CI*: 0.79–0.92) but positively associated with MUNO (*OR* = 1.16, 95% *CI*: 1.08–1.24). **Conclusions:** The Rice–Vegetable–Pork pattern was associated with more favorable obesity-related metabolic phenotypes, whereas energy-dense, animal-derived patterns were associated with higher odds of obesity phenotypes; the Soybeans–Tubers–Grains pattern showed mixed associations.

## 1. Introduction

According to the United Nations World Social Report 2023, population aging is accelerating globally. By 2050, the number of older adults worldwide is projected to double, and China is expected to become one of the most aged countries in Asia [1]. Aging is accompanied by insufficient physical activity, unhealthy dietary habits, and a decrease in basal metabolic rate and nutritional requirements, making middle-aged and older adult populations highly susceptible to obesity and related metabolic abnormalities [2,3]. Obesity is not only an independent health issue but also often coexists with metabolic disorders such as hypertension, dyslipidemia, and hyperglycemia, significantly increasing the incidence and mortality risks of cardiovascular diseases, type 2 diabetes, and various cancers [4,5,6].

In recent years, researchers have further subdivided obesity into four metabolic phenotypes based on body mass index (BMI) and metabolic status: metabolically healthy non-obese (MHNO), metabolically unhealthy non-obese (MUNO), metabolically healthy obese (MHO), and metabolically unhealthy obese (MUO) [7,8]. Among these, MHO was once regarded as “benign obesity”; however, longitudinal studies have shown that MHO is a transient state with a dynamic risk of transitioning to MUO, suggesting that this phenotype may represent a transitional phase of metabolic dysfunction [9,10]. Therefore, early identification of modifiable factors influencing different obesity-related metabolic phenotypes is crucial for achieving precision prevention and intervention.

As one of the modifiable lifestyle factors, diet is closely associated with obesity and metabolic health [11]. Compared with analyzing individual nutrients or foods in isolation, dietary patterns better reflect the overall dietary structure and its synergistic effects, thus gaining increasing attention in chronic disease research [12,13]. Existing studies have shown significant associations between different dietary patterns and metabolic health outcomes [14]. However, most of the existing evidence focuses on overall metabolic health without considering obesity, and the association between dietary patterns and different obesity-related metabolic phenotypes remains unclear in middle-aged and older Chinese adults.

Therefore, this study utilizes national data to analyze dietary habits of Chinese adults ≥45 years, explore the relationship between dietary patterns and different obesity-related metabolic phenotypes, and identify key dietary factors influencing the health status of this population. This is crucial for the early prevention and control, precise treatment, and personalized intervention of chronic diseases.

## 2. Materials and Methods

### 2.1. Participants

Data were derived from the 2015 China Adults National Chronic Disease and Nutrition Surveillance (CANCDNS), a multistage stratified cluster sample across 31 provincial-level administrative divisions. Adults aged ≥45 years with complete basic information, dietary survey data and fasting biochemical results were eligible. Participants with missing key identifiers and implausible energy intake (<800 or >5000 kcal/day) were excluded [15]. A total of 32,091 individuals were included. The protocol was approved by the Ethics Review Committee of the Chinese Center for Disease Control and Prevention (No. 201519-B, 15 June 2015). All participants provided written informed consent.

### 2.2. Questionnaire Survey and Dietary Pattern Analysis

Data collection was conducted through one-on-one household surveys to gather basic information and dietary intake data of the participants. For dietary data collection, individual food intake was assessed using three consecutive 24 h dietary recalls, which recorded all foods consumed by each participant except major condiments such as cooking oil and salt. At the same time, the household weighing inventory method was used to measure the total consumption of major condiments at the household level during the same 3-day survey period. Individual condiment intake was then estimated based on household condiment consumption, combined with each participant’s dietary records and shared household meal information.

Based on the Chinese Food Composition Table [16], the average daily intake of various food groups and total energy per person was calculated. Foods were classified into predefined food groups according to their main ingredients and composition. Dietary pattern analysis was performed based on the average daily intake of each food group. A total of 19 food groups were included for dietary pattern extraction. Exploratory factor analysis (EFA) was employed to extract dietary patterns. The Kaiser–Meyer–Olkin (KMO) test and Bartlett’s test of sphericity were used to assess the suitability of data for factor analysis. Principal component analysis (PCA) with varimax rotation was applied to perform EFA and extract dietary patterns. The number of dietary patterns was determined based on an eigenvalue >1 and the scree plot, while the main food groups of each dietary pattern were identified by an absolute factor loading >0.3. A higher factor loading indicates a stronger tendency of participants to consume the corresponding food. For the dietary data of Chinese population aged 45 and above in this study, the KMO test yielded a statistic of 0.704, and Bartlett’s test of sphericity showed *p* < 0.001, indicating strong correlations among food groups and confirming the data’s suitability for factor analysis. A total of four dietary patterns were extracted based on the eigenvalue >1 and the scree plot.

### 2.3. Anthropometric Indicator Measurement and Blood Index Detection

Blood glucose was measured using the glucose oxidase method; triglycerides were determined via the glycerol phosphate oxidase-4-chlorophenol method; and high-density lipoprotein cholesterol (HDL-C) was assayed by the direct method. Anthropometric measurements were performed by trained staff using standardized procedures. Body weight and height were measured with participants wearing light clothing and no shoes, and BMI was calculated as weight (kg) divided by height squared (m^2^). Waist circumference (WC) was measured at the midpoint between the lowest rib and the iliac crest. Blood pressure was measured in a seated position after at least 5 min of rest using a calibrated device; the mean of two readings was used for analysis.

Operational definitions were as follows: ① Smoking and Alcohol Consumption: Smoking was defined as an affirmative response to the question “Do you currently smoke?” [17]. Alcohol consumption was defined as drinking at least once per week [17]. ② Adequate Physical Activity: adults engaging in at least 150–300 min of moderate-intensity aerobic activity or 75–150 min of vigorous-intensity activity per week [18].

### 2.4. Assessment of the Obesity-Related Metabolic Phenotypes

The obesity-related metabolic phenotypes were categorized into four groups on the basis of the presence of obesity and metabolic abnormalities as follows: MHNO, MUNO, MHO, and MUO. Metabolic abnormalities were defined as meeting three or more of the following criteria [19]: (1) waist circumference (WC) > 102 cm (males) or >88 cm (females); (2) triglycerides (TG) ≥ 1.70 mmol/L; (3) high-density lipoprotein cholesterol (HDL-C) < 1.30 mmol/L (females) or <1.04 mmol/L (males); (4) systolic blood pressure (SBP) ≥ 130 mmHg and/or diastolic blood pressure (DBP) ≥ 85 mmHg; (5) fasting plasma glucose (FPG) ≥ 6.1 mmol/L. Conversely, individuals not meeting these criteria were considered metabolically normal. Obesity was defined according to age-specific Chinese BMI cut-offs. For participants aged 45–64 years, obesity was defined as BMI ≥ 28 kg/m^2^ [20]. For those aged ≥65 years, obesity was defined as BMI ≥ 27 kg/m^2^ [21].

### 2.5. Quality Control

To ensure the quality of monitoring, the national project working group developed and implemented a quality control protocol. Four uniform standards were adopted during the monitoring process: unified protocols, manuals, and questionnaires; unified training and assessment; unified equipment and reagents; and unified data entry and cleaning.

### 2.6. Statistical Analysis

Data cleaning and analysis were performed using SPSS 26.0 and R 4.5.1. Descriptive statistics of baseline characteristics were presented as frequencies and percentages, and difference tests were conducted using the Chi-square test. After adjusting for confounding factors including total energy, sex, age, educational level, physical activity, urban–rural residence, region, marital status, smoking status, and alcohol consumption, multinomial logistic regression was used to analyze the association between dietary patterns and different obesity-related metabolic phenotypes. The results were presented as odds ratios (*ORs*) with 95% confidence intervals (95% *CIs*). In addition, subgroup analyses were conducted by sex. All statistical tests were two-tailed, and a *p*-value < 0.05 was considered statistically significant. In addition, to characterize the nutrient profiles of the identified dietary patterns, total energy and nutrient intakes were described across tertiles of four dietary pattern scores. Province-specific mean factor scores for each dietary pattern were also calculated, and the pattern with the highest mean score within a province was defined as the dominant dietary pattern to describe the geographic distribution of dietary patterns across China.

## 3. Results

### 3.1. Characteristics of the Participants

A total of 32,091 participants were included, of whom 48.7% were men and 51.3% were women (Table 1). Overall, 48.2% were aged 45–59 years and 51.8% were aged ≥60 years. Regarding obesity-related metabolic phenotypes, 63.7% were classified as MHNO, 11.2% as MUNO, 10.8% as MHO and 14.4% as MUO. Baseline characteristics differed significantly across phenotypes in terms of age, sex, urban–rural residence, region, education, marital status, smoking and alcohol consumption (all *p* < 0.05).

### 3.2. Dietary Pattern Analysis

The cumulative variance contribution rate of the four dietary patterns extracted in this study was 34.1%, and the dietary patterns were named based on the food groups corresponding to factors with high factor loading values: (1) Pattern 1, The Rice–Vegetable–Pork pattern, was characterized by higher intakes of rice, vegetables, pork, poultry and aquatic products, and lower intakes of wheat and other grains/legumes; (2) Pattern 2, the Fruit–Egg–Dairy pattern, involved relatively high consumption of fruits, eggs, dairy products, nuts, and mushrooms and algae; (3) Pattern 3, the Red Meat–Offal–Snack pattern, included increased intakes of beef, mutton, animal offal and snacks; (4) Pattern 4, the Soybeans–Tubers–Grains pattern, featured higher consumption of soybeans, tubers, and other grains and miscellaneous beans, as shown in Table 2.

### 3.3. Nutrient Characteristics of the Four Dietary Patterns

Across tertiles of pattern scores (Table 3), Pattern 4 showed higher dietary fiber and magnesium intakes, whereas Pattern 1 was characterized by higher intakes of several micronutrients (e.g., vitamins A/C, Ca and K). Patterns 2 and 3 exhibited higher total energy and fat intakes. Na intake was high across all patterns, with only modest differences across score categories.

### 3.4. Geographic Distribution of Dietary Patterns Across Provinces

**Figure 1:**
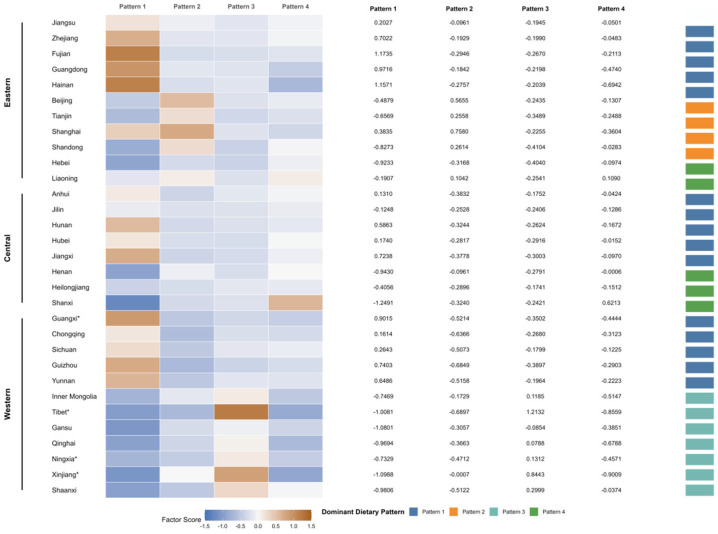
Provincial distribution of mean dietary pattern scores and dominant dietary patterns among middle-aged and older Chinese adults. Heatmap cells show mean scores for Patterns 1–4 by province; Exact numeric values are shown in the adjacent panel, * denotes autonomous regions.

Beyond nutrient profiles, we further examined how these dietary patterns are distributed geographically across China (Figure 1). The mean dietary pattern scores and the dominant pattern varied markedly across China’s 31 provincial-level administrative divisions, indicating clear geographic clustering. In Eastern China, Pattern 1 predominated in several south-eastern coastal provinces. In contrast, Pattern 2 was mainly observed in municipalities and economically developed areas. In Central China, Pattern 1 was dominant in Anhui, Jilin, Hubei, and Hunan, whereas Henan and Heilongjiang tended to show higher scores for Pattern 4. In Western China, Pattern 3 clustered in north-western/plateau regions.

### 3.5. Association Between Dietary Patterns and Obesity-Related Metabolic Phenotypes

**Figure 2:**
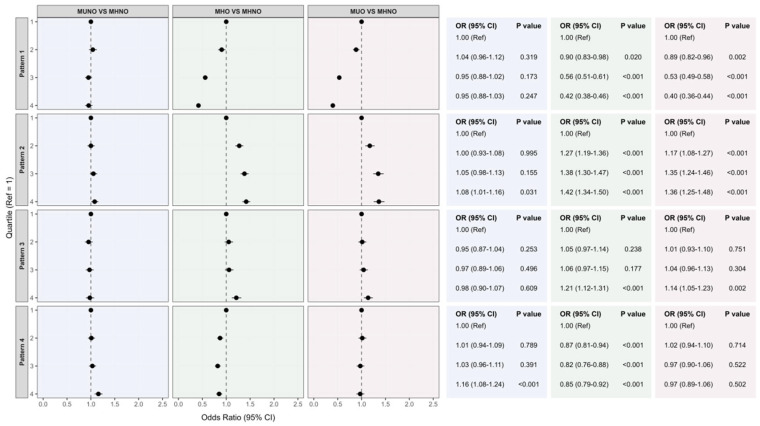
Associations between quartiles of dietary patterns and different obesity phenotypes.

Figure 2 shows the associations between quartiles of the four dietary patterns and obesity-related metabolic phenotypes after adjustment for key covariates. The Rice–Vegetable–Pork pattern was inversely associated with MHO (OR = 0.42, *p* < 0.001) and MUO (OR = 0.40, *p* < 0.001), with no significant association observed for MUNO. In contrast, the Fruit–Egg–Dairy pattern was positively associated with MHO (OR = 1.42, *p* < 0.001) and MUO (OR = 1.36, *p* < 0.001), and the Red Meat–Offal–Snack pattern was also positively associated with MHO (OR = 1.21, *p* < 0.01) and MUO (OR = 1.14, *p* < 0.01). The Soybeans–Tubers–Grains pattern was inversely associated with MHO (OR = 0.85, *p* < 0.001) and positively associated with MUNO (OR = 1.16, *p* < 0.001).

### 3.6. Association Between Dietary Pattern Scores and Obesity-Related Metabolic Phenotypes by Sex

**Figure 3:**
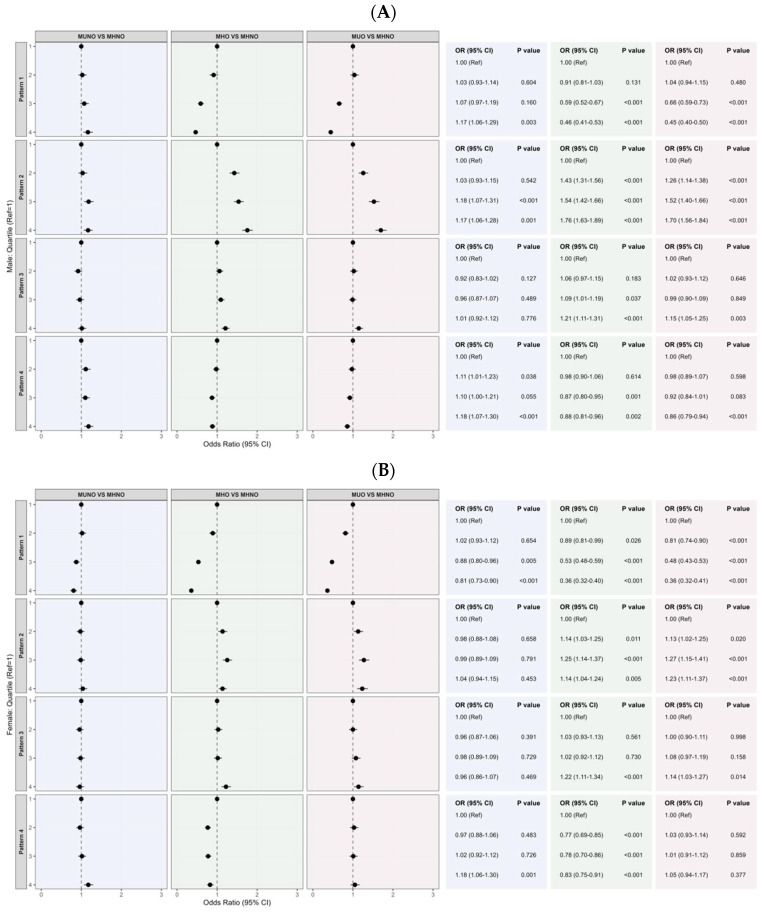
Associations between quartiles of dietary patterns and different obesity phenotypes stratified by sex. Note: Models were adjusted for total energy, age, educational level, physical activity, urban–rural residence, region, marital status, smoking status, and alcohol consumption. (**A**): Male; (**B**): Female. Pattern 1: Rice–Vegetable–Pork pattern; Pattern 2: Fruit–Egg–Dairy pattern; Pattern 3: Red Meat–Offal–Snack pattern; Pattern 4: Soybeans–Tubers–Grains pattern.

Stratified analyses by sex revealed notable differences in the associations between dietary patterns and obesity-related metabolic phenotypes (Figure 3). In males, Pattern 2 showed a particularly strong positive association with the risk of obesity phenotypes (MHO and MUO). In contrast, among females, Pattern 1 was significantly associated with a lower risk of MUNO, an association not observed in males. Additionally, Pattern 4 was mainly protective for MHO in females, with only a small reduction in MUO among males.

## 4. Discussion

Using nationally representative CANCDNS data, we examined how empirically derived dietary patterns relate to obesity-related metabolic phenotypes among Chinese adults aged ≥45 years. Approximately one quarter of participants were obese (MHO or MUO), and more than half of those with obesity were metabolically unhealthy, highlighting a substantial cardiometabolic burden in later life. Given evidence that MHO is often a transient state with progression to MUO over time [22], identifying modifiable determinants that may delay metabolic deterioration is clinically relevant. Phenotype groups differed by key sociodemographic and lifestyle factors, underscoring the need for careful confounding control and targeted prevention strategies. Together, these results suggest that metabolic abnormalities are common even without obesity, reinforcing the importance of diet as a potentially modifiable factor to prevent worsening metabolic health.

Obesity-related metabolic phenotypes, defined jointly by adiposity and metabolic status, provide a clinically meaningful framework to stratify cardiometabolic risk. In the present study, MHNO individuals accounted for 63.7% of participants, whereas the proportions of MUNO, MHO and MUO were 11.2%, 10.8%, and 14.4%, respectively. Although the distribution was broadly consistent with prior reports, the relatively high proportion of MUO underscores persistent challenges in metabolic risk control among aging Chinese adults. Prospective evidence indicates that MHO frequently progresses to MUO and is associated with elevated long-term cardiovascular risk, making prevention of metabolic deterioration a public health priority [23].

Using data-driven factor analysis, we identified four dietary patterns that captured distinct combinations of staple foods and accompanying food groups. These patterns plausibly reflect China’s ongoing nutrition transition, in which traditional plant-forward diets coexist with greater availability of animal-derived and processed foods—an epidemiologic context relevant to rising cardiometabolic risk.

Nutrient profiles across pattern–score categories support biological plausibility: plant-forward patterns were more fiber- and micronutrient-dense, whereas animal-derived/processed patterns were more energy-, fat-, and sodium-dense. These contrasts are relevant because higher energy density and sodium intake, together with an unfavorable fat profile, may contribute to insulin resistance, hypertension and dyslipidaemia [24], whereas fiber- and micronutrient-rich diets may improve glycemic regulation and satiety and support gut microbiome-related metabolic pathways.

Our findings also underscore pronounced geographic clustering of dietary patterns. Pattern 1 predominated in southern and south-western rice-growing PLADs (provincial-level administrative divisions), whereas Pattern 4 was more common in northern and parts of central China, where wheat and coarse grains are traditional staples. Pattern 2 was concentrated in large municipalities and more economically developed eastern PLADs, likely reflecting higher availability and adoption of dairy, fruit and processed foods with increasing urbanicity. Pattern 3 predominated in north-western and plateau pastoral PLADs, in line with historically higher intakes of red meat and offal in these regions. Together, these regional contrasts mirror the well-described nutrition transition in China and may contribute to geographic disparities in obesity and metabolic risk [22,25].

Overall, our findings are broadly consistent with international evidence linking plant-forward dietary patterns to better metabolic health and Western-style patterns to higher cardiometabolic risk. Studies across diverse settings suggest that patterns rich in whole grains, vegetables and fruits are associated with healthier metabolic profiles, whereas patterns high in red/processed meats and energy-dense foods are associated with obesity and metabolic abnormalities [26,27]. Consistently, evidence from China indicates that grain–vegetable-based patterns with moderate meat intake are generally associated with lower obesity and cardiometabolic risk [28].

Pattern 1 was associated with markedly lower odds of MHO and MUO (Q4 vs. Q1: *OR* = 0.42, 95% *CI*: 0.38–0.46 for MHO; *OR* = 0.40, 95% *CI*: 0.36–0.44 for MUO). Rice contributed strongly to this pattern (high factor loading), but the observed association likely reflects the overall dietary context, including higher vegetable intake and a comparatively lower energy density. This pattern aligns with recommendations emphasizing plant foods while limiting energy-dense, highly processed items [29]. Its protective association may relate to a more favorable nutrient profile that supports insulin sensitivity and lipid metabolism [30].

Pattern 2 was associated with higher odds of both MHO and MUO (Q4 vs. Q1: *OR* = 1.42, 95% *CI*: 1.34–1.50 for MHO; *OR* = 1.36, 95% *CI*: 1.25–1.48 for MUO). This finding does not imply that all fruit or dairy intake is harmful; rather, the overall pattern may reflect co-occurring dietary behaviors and food choices associated with a less favorable nutrient profile. In contemporary Chinese diets, higher fruit and dairy consumption may sometimes coincide with greater intake of sweetened beverages or flavored dairy products, which may contribute to higher added sugar intake and other adverse dietary characteristics. Because total energy intake was adjusted for in our models, these associations should not be interpreted as being explained solely by energy intake [31].

Similarly, Pattern 3 was associated with higher odds of MHO and MUO (Q4 vs. Q1: *OR* = 1.21, 95% *CI*: 1.12–1.31 for MHO; *OR* = 1.14, 95% *CI*: 1.05–1.23 for MUO), consistent with its energy-dense profile and higher sodium intake. High intakes of red meat, offal and packaged snacks may contribute to excess energy and sodium intake and a less favorable lipid profile, which are plausibly linked to insulin resistance and chronic inflammation [32].

Pattern 4 showed mixed associations—lower odds of MHO but higher odds of MUNO (Q4 vs. Q1: *OR* = 0.85 and 1.16)—which may reflect heterogeneity in food processing/preparation, residual confounding, or potential reverse causation in cross-sectional data. Public health messaging that promotes minimally processed legumes and tubers prepared by boiling/steaming, rather than frying or heavy salting, may help maximize potential metabolic benefits [33]. Future studies distinguishing cooking methods (e.g., steaming/boiling vs frying) and added salt/oil at the dish level may clarify whether the observed heterogeneity reflects preparation-related factors or behavioral changes after diagnosis.

Sex-stratified findings may reflect adipose distribution and metabolic responses. Men tend to accumulate more visceral abdominal fat, which is closely linked to insulin resistance and predicts progression from metabolically healthy to unhealthy obesity [23], potentially amplifying the adverse associations of the modern high-protein/processed pattern with MHO and MUO. In contrast, women more commonly store subcutaneous fat and may derive greater metabolic benefit from traditional balanced and fiber-rich plant-forward diets, consistent with intervention evidence in metabolically healthy obesity [34] and population-level observations in China [28]. Moreover, sex differences in dietary behaviors and long-term weight-gain trajectories may further modify diet-phenotype associations [35].

This study has several strengths. First, we used a large, nationally representative sample, improving generalizability to Chinese adults aged ≥45 years. Second, diet was assessed using three consecutive 24 h recalls combined with household condiment weighing, strengthening the assessment of sodium- and oil-related exposures in Chinese cooking contexts. Third, we integrated food-based patterns with nutrient profiles and province-level geographic clustering, enabling more actionable and region-tailored interpretation. Finally, multinomial modeling allowed simultaneous comparison across multiple obesity-related metabolic phenotypes with adjustment for key covariates, and sex-stratified analyses provided additional insight into heterogeneity.

There are several limitations in this study. First, the cross-sectional design precludes causal inference between dietary patterns and obesity-related metabolic phenotypes, only suggesting associations. Second, dietary data were collected using three consecutive 24 h dietary recalls and weighing inventory methods. Despite rigorous quality control measures, recall bias and day-to-day variations in diet could not be entirely eliminated [36]. Third, the dietary pattern analysis was based on food groups without further distinguishing food processing methods, such as whole fruits versus fruit juices, whole grains versus refined grains, or baked versus fried preparations, which may substantially influence metabolic outcomes [37]. Fourth, because there is no fully unified definition of obesity-related metabolic phenotypes in China, we classified phenotypes using a commonly applied set of metabolic abnormality criteria. However, alternative thresholds across studies may introduce some misclassification and limit cross-study comparability.

## 5. Conclusions

Overall, using nationally representative data, we identified four major dietary patterns with distinct nutrient profiles and geographic distributions among Chinese adults aged ≥45 years and examined their associations with obesity-related metabolic phenotypes. Two plant-rich patterns, characterized by higher intakes of rice, vegetables, legumes, tubers and coarse grains, were associated with lower odds of MHO and/or MUO, whereas excessive intake of the “animal-derived and processed food pattern”—characterized by high consumption of animal products, sugary dairy products, and snacks, and reflecting an energy-dense dietary profile—was associated with increased odds of metabolically unhealthy phenotypes. These associations were generally consistent across sex subgroup, although the strength of associations varied. Our findings support maintaining and optimizing traditional plant-rich Chinese dietary structures while limiting further spread of snack-rich and sugar-sweetened dairy patterns, particularly in rapidly urbanizing and north-western regions, to help reduce the growing burden of obesity-related metabolic disorders in China.

## Figures and Tables

**Table 1:** Baseline characteristics of participants by obesity-related metabolic phenotypes [*n*(%)].

Characteristics	Total (*n* = 32,091)	MHNO (*n* = 20,426)	MUNO (*n* = 3586)	MHO (*n* = 3454)	MUO (*n* = 4625)	*p*-Value
Age (years)						
45–59	15,483	10,176 (49.8)	1084 (30.2)	1905 (55.2)	2318 (50.1)	<0.001
60–74	14,248	8824 (43.2)	2043 (57.0)	1337 (38.7)	2044 (44.2)	
75–	2360	1426 (7.0)	459 (12.8)	212 (6.1)	263 (5.7)	
Sex						
Male	15,644	11,040 (54.0)	1183 (33.0)	1884 (54.5)	1537 (33.2)	<0.001
Female	16,447	9386 (46.0)	2403 (67.0)	1570 (45.5)	3088 (66.8)	
Urban and rural areas						
City	13,079	7522 (36.8)	1577 (44.0)	1677 (48.6)	2303 (49.8)	<0.001
Rural	19,012	12,904 (63.2)	2009 (56.0)	1777 (51.4)	2322 (50.2)	
Region ^#^						
Eastern	12,272	7332 (35.9)	1469 (41.0)	1497 (43.4)	1974 (42.7)	<0.001
Central	9544	6084 (29.8)	1148 (32.0)	957 (27.7)	1355 (29.3)	
Western	10,255	7001 (34.3)	965 (27.0)	998 (28.9)	1291 (28.0)	
Education level						
Primary school and below	18,787	12,160 (59.5)	2295 (64.0)	1763 (51.0)	2569 (55.5)	<0.001
Junior or senior high school	12,131	7547 (36.9)	1151 (32.1)	1547 (44.8)	1886 (40.8)	
College degree or above	1173	719 (3.5)	140 (3.9)	144 (4.2)	170 (3.7)	
Marital status						
Married	29,830	19,081 (93.4)	3192 (89.0)	3263 (94.5)	4294 (92.8)	<0.001
Others	2261	1345 (6.6)	394 (11.0)	191 (5.5)	331 (7.2)	
Smoking						
Yes	8061	6030 (29.5)	653 (18.2)	680 (19.7)	698 (15.1)	<0.001
No	24,028	14,394 (70.5)	2933 (81.8)	2774 (80.3)	3927 (84.9)	
Drinking						
Yes	6659	4770 (23.4)	551 (15.4)	723 (20.9)	615 (13.3)	<0.001
No	25,432	15,656 (76.6)	3035 (84.6)	2731 (79.1)	4010 (86.7)	

Note: Chi-square test was used for inter-group comparisons. ^#^ The 31 provincial-level administrative divisions were classified into three geographic regions: Eastern: Beijing Municipality, Tianjin Municipality, Hebei Province, Liaoning Province, Shanghai Municipality, Jiangsu Province, Zhejiang Province, Fujian Province, Shandong Province, Guangdong Province, and Hainan Province; Central: Shanxi Province, Jilin Province, Heilongjiang Province, Anhui Province, Jiangxi Province, Henan Province, Hubei Province, and Hunan Province; Western: Inner Mongolia Autonomous Region, Guangxi Zhuang Autonomous Region, Chongqing Municipality, Sichuan Province, Guizhou Province, Yunnan Province, Tibet Autonomous Region, Shaanxi Province, Gansu Province, Qinghai Province, Ningxia Hui Autonomous Region, and Xinjiang Uygur Autonomous Region.

**Table 2:** Factor loadings of food groups in four dietary patterns.

	Pattern 1: Rice–Vegetable–Pork Pattern	Pattern 2: Fruit–Egg–Dairy Pattern	Pattern 3: Red Meat–Offal–Snack Pattern	Pattern 4: Soybeans–Tubers–Grains Pattern
Rice and its products	**0.764**	−0.184	−0.047	0.004
Wheat and its products	**−0.694**	0.117	0.060	−0.022
Other grains and legumes	**−0.433**	−0.060	−0.123	**0.384**
Tubers	−0.186	−0.152	0.145	**0.561**
Vegetables	**0.438**	0.133	−0.092	0.281
Mushrooms and algae	0.107	**0.348**	0.011	−0.045
Pickled vegetables	0.001	0.005	0.006	−0.064
Fruits	0.034	**0.638**	0.080	0.116
Pork	**0.611**	0.030	−0.020	0.042
Beef and mutton	−0.135	0.105	**0.669**	−0.153
Animal offal	0.137	−0.079	**0.588**	0.104
Poultry meat	**0.329**	0.144	0.162	−0.061
Aquatic products	**0.442**	0.219	0.046	−0.039
Eggs	−0.062	**0.563**	−0.136	0.056
Dairy products	−0.100	**0.525**	−0.136	0.056
Soybeans	0.119	0.073	−0.036	**0.601**
Nuts	0.096	**0.403**	0.107	0.228
Snacks	−0.073	0.007	**0.590**	−0.038
Sugary beverages	0.155	0.052	0.286	0.217

Note: Absolute factor loading > 0.3 is shown in bold.

**Table 3:** Nutrient characteristics across tertiles of dietary pattern scores among Chinese adults aged ≥45 years [median (P25, P75)].

**Nutritional Components**	**Pattern 1**			**Pattern 2**		
	**Q1 (*n* = 10,695)**	**Q2 (*n* = 10,696)**	**Q3 (*n* = 10,700)**	**Q1 (*n* = 10,696)**	**Q2 (*n* = 10,696)**	**Q3 (*n* = 10,699)**
Energy (kcal/d)	1646.6 (1305.3–2054.1)	1466.2 (1178.5–1827.2)	1887.3 (1541.3–2324.9)	1551.4 (1211.1–1950.0)	1651.36 (1308.8–2064.55)	1791.9 (1450.6–2213.9)
Protein (g/d)	46.0 (35.3–59.3)	42.4 (31.9–55.5)	59.5 (47.1–75.6)	39.5 (29.8–51.1)	48.85 (37.96–62.03)	60.1 (47.6–76.4)
Fat (g/d)	53.3 (36.7–75.6)	60.6 (43.5–84.0)	79.4 (58.2–107.1)	57.3 (38.6–83.4)	63.71 (44.77–89.55)	70.6 (51.2–96.3)
Carbohydrates (g/d)	240.9 (185.2–307.9)	180.4 (140.2–232.6)	224.0 (171.1–291.3)	208.3 (155.5–276.0)	208.03 (156.9–275.42)	221.0 (173.1–286.3)
Protein (% of Energy)	11.3 (9.9–12.9)	11.6 (9.5–13.9)	12.6 (10.4–15.3)	10.2 (8.7–11.9)	11.81 (10.15–13.8)	13.4 (11.5–15.7)
Fat (% of Energy)	30.0 (23.0–38.0)	38.1 (30.0–47.0)	38.7 (31.0–47.0)	34.5 (25.2–44.7)	36.17 (27.44–45.29)	36.1 (28.8–43.8)
Carbohydrate (% of Energy)	60.2 (52.1–67.9)	50.8 (42.4–59.0)	48.5 (39.6–57.2)	56.0 (46.1–65.4)	52.68 (43.17–61.88)	51.5 (43.0–59.3)
Cholesterol (mg/d)	29.4 (4.0–75.4)	79.4 (38.9–139.1)	172.5 (104.4–262.5)	56.0 (15.0–117.0)	89.24 (31.18–176.85)	125.4 (54.7–244.8)
Dietary fiber (g/d)	9.2 (6.6–12.6)	7.1 (4.9–10.2)	7.9 (5.7–11.3)	6.5 (4.7–9.2)	7.87 (5.65–10.94)	10.2 (7.4–14.1)
Vitamin A (μg/d)	170.2 (80.7–313.7)	258.4 (137.0–450.3)	408.9 (220.5–691.8)	166.0 (69.0–372.7)	231.14 (129.38–438.71)	375.3 (231.9–619.3)
Vitamin C (mg/d)	51.7 (31.4–79.3)	61.3 (39.2–91.1)	83.2 (55.0–118.4)	57.4 (34.3–87.0)	61.7 (38.75–92.59)	75.4 (48.9–110.8)
Vitamin E (mg/d)	29.8 (19.1–47.5)	26.8 (17.2–41.3)	24.3 (14.8–37.7)	23.9 (14.0–39.1)	27.03 (17.13–42.26)	29.3 (19.7–43.4)
Ca (mg/d)	259.9 (188.8–352.8)	266.6 (189.9–377.0)	344.7 (253.2–467.7)	225.5 (163.3–311.3)	276.5 (208.49–367.39)	379.4 (282.6–508.3)
K (mg/d)	1274.3 (972.4–1637.7)	1165.4 (880.9–1545.3)	1478.3 (1170.1–1889.0)	1066.3 (815.8–1367.1)	1262.43 (1001.26–1589.6)	1633.8 (1302.7–2035.5)
Na (mg/d)	6477.2 (4208.8–9911.4)	5698.7 (3788.1–8612.4)	6196.0 (4181.8–9163.7)	6160.6 (3989.1–9515.5)	6337.1 (4158.21–9413.92)	5876.2 (3980.9–8651.1)
Mg (mg/d)	238.6 (181.8–310.0)	194.3 (150.4–254.9)	238.2 (191.2–299.3)	192.3 (149.1–247.2)	219.21 (172.84–280.25)	264.3 (208.4–334.2)
**Nutritional Components**	**Pattern 3**			**Pattern 4**		
	**Q1 (*n* = 10,696)**	**Q2 (*n* = 10,695)**	**Q3 (*n* = 10,700)**	**Q1 (*n* = 10,695)**	**Q2 (*n* = 10,696)**	**Q3 (*n* = 10,700)**
Energy (kcal/d)	1640.2 (1304.2–2049.1)	1558.2 (1233.4–1945.7)	1799.2 (1443.0–2242.5)	1524.7 (1199.9–1933.4)	1613.1 (1285.3–1990.7)	1858.9 (1507.4–2285.5)
Protein (g/d)	48.3 (36.9–62.0)	44.1 (32.9–57.7)	55.8 (42.4–72.3)	44.8 (33.3–60.0)	46.1 (35.4–59.1)	56.4 (43.9–71.5)
Fat (g/d)	63.4 (44.7–88.9)	60.3 (41.6–84.7)	68.3 (47.9–97.2)	61.5 (42.4–87.8)	63.2 (44.7–87.7)	67.6 (46.7–94.2)
Carbohydrates (g/d)	210.5 (158.7–278.1)	200.8 (154.0–263.1)	228.0 (175.6–298.0)	185.7 (142.2–245.3)	204.3 (158.7–265.5)	252.1 (196.7–318.5)
Protein (% of Energy)	11.7 (10.0–13.8)	11.2 (9.4–13.3)	12.3 (10.3–14.8)	11.8 (9.7–14.1)	11.4 (9.7–13.5)	12.0 (10.3–14.3)
Fat (% of Energy)	36.0 (27.4–45.1)	35.8 (27.0–44.9)	35.2 (27.1–43.9)	37.4 (28.6–46.8)	36.4 (28.0–45.0)	33.3 (25.3–41.8)
Carbohydrate (% of Energy)	53.1 (43.8–62.1)	53.7 (44.4–62.8)	52.8 (43.5–61.7)	50.8 (41.5–60.2)	52.8 (43.9–61.6)	55.6 (46.6–64.5)
Cholesterol (mg/d)	70.1 (21.3–160.1)	69.1 (21.3–142.6)	120.6 (57.3–220.2)	96.8 (39.5–189.7)	83.4 (29.6–170.3)	78.0 (24.0–170.9)
Dietary fiber (g/d)	8.3 (5.8–11.9)	7.3 (5.1–10.3)	8.7 (6.1–12.3)	5.9 (4.3–8.3)	7.6 (5.8–10.2)	11.2 (8.4–15.1)
Vitamin A (μg/d)	278.3 (150.9–497.4)	225.6 (106.5–422.1)	283.4 (136.9–550.7)	213.5 (105.7–399.7)	265.4 (133.6–489.1)	315.8 (159.3–580.9)
Vitamin C (mg/d)	66.5 (41.3–99.0)	59.6 (36.8–89.6)	68.0 (41.9–102.6)	44.2 (27.4–68.5)	65.4 (44.6–94.38)	87.8 (59.0–123.8)
Vitamin E (mg/d)	26.4 (16.9–40.2)	25.7 (16.0–39.9)	28.8 (18.0–45.3)	23.0 (13.8–37.4)	25.5 (16.6–39.3)	32.0 (21.5–47.2)
Ca (mg/d)	296.0 (218.3–403.0)	263.2 (185.2–371.1)	306.3 (218.4–429.3)	231.1 (164.6–330.7)	277.3 (207.7–366.4)	363.2 (272.3–487.0)
K (mg/d)	1270.8 (982.7–1641.4)	1197.0 (902.0–1554.2)	1468.5 (1128.7–1890.9)	1071.9 (818.6–1399.2)	1238.1 (990.1–1552.4)	1637.9 (1317.8–2047.5)
Na (mg/d)	6121.1 (4050.9–9011.1)	5922.4 (3910.5–8995.9)	6325.7 (4157.4–9563.7)	5946.2 (3886.4–9188.2)	6025.4 (4020.9–9044.2)	6357.7 (4217.9–9326.6)
Mg (mg/d)	228.1 (176.0–296.3)	206.0 (158.7–266.4)	238.9 (186.6–306.1)	182.7 (141.8–232.6)	212.1 (172.6–262.5)	285.3 (231.2–353.8)

Abbreviations: Ca, calcium; K, potassium; Na, Sodium; Mg, magnesium.

## Data Availability

Data for this study were derived from the 2015 China Adults National Chronic Disease and Nutrition Surveillance (CANCDNS). The datasets are not publicly available; access is subject to approval by the data owner/institution. Data may be available from the corresponding author upon reasonable request and with appropriate permission.
